# Tumor ablation induced anti-tumor immunity: destruction of the tumor *in situ* with the aim to evoke a robust anti-tumor immune response

**DOI:** 10.1007/s10555-023-10150-x

**Published:** 2023-11-11

**Authors:** Yona Keisari, Itzhak Kelson

**Affiliations:** 1 Department of Clinical Microbiology and Immunology, Faculty of Medicine, Tel Aviv University, 6997801 Tel Aviv, Israel; 2 Sackler Faculty of Exact Sciences, School of Physics and Astronomy, Tel Aviv University, 6997801 Tel Aviv, Israel

**Keywords:** Tumor ablation, *In situ* tumor destruction, Anti-tumor immunity, Immunomanipulation, Cancer, Immunotherapy, Anti-metastatic effect

## Anti-tumor immunity

Immunotherapy of cancer is a major goal since 1891 when William Coley started an experimental treatment of cancer patients with bacterial-derived products. He introduced, currently known, as the first non-specific immunostimulatory approach, claiming that the beneficial effect is a result of boosting the patient’s systemic response against the tumor [1]. This effort is constantly growing in the last 130 years, but it is important to note that most of the current immunotherapy modalities rely on boosting the anti-tumor immune response activity of the patient himself.

The role of the immune response in tumor development and treatment is a very complicated issue. There are many elements involved such as the variety of tumor types, the organism genetics, the complexity of the immune response, metabolism, age, and the microbiome to name a few.

The goal of therapeutic cancer vaccines is to induce tumor regression, eradicate minimal residual disease, establish lasting antitumor memory, and avoid non-specific or adverse reactions [2]. Different strategies based on peptides are available for cancer vaccines. The peptides selected for cancer vaccine development can be classified into two main types: tumor-associated antigens (TAAs) and tumor-specific antigens (TSAs), which are captured, internalized, processed, and presented by antigen-presenting cells (APCs) to cell-mediated immunity. Peptides loaded onto MHC class I are recognized by a specific TCR of CD8 + T cells, which are activated to exert their cytotoxic activity against tumor cells presenting the same peptide-MHC-I complex.

Yet even though about 70 years elapsed since the discovery of tumor antigens [3], the success of therapeutic cancer vaccines has been very limited and variable, particularly in advanced cancer patients. It mainly resulted from the heterogeneity of the tumor and its microenvironment, the presence of immunosuppressive cells and molecules, and the potential for tumor escape mechanisms [4]. Additionally, the effectiveness of these therapies may be limited by the variability of the patient’s immune system response and the difficulty in identifying appropriate antigens for each patient.

In this commentary, we will portray the interrelationship between two multifaceted fields—tumor ablation and anti-tumor immunity. Due to the large amount of literature on this topic, most of the cited articles are reviews.

## The tumor as its own vaccine

The failure to develop effective specific tumor vaccines to block the development of metastatic tumors reverted the attention to an observation, which is now 70 years old, “the abscopal effect.” This effect states that irradiation of a tumor site can cause the decrease in size of distant tumor foci. It was originally defined by Mole “at a distance from the irradiated volume but within the same organism” [5] and related to lymphocyte function by Nobler [6]. This observation was neglected for almost 20 years due to poor information and understanding of the immune response, although several studies which tried to tie radiotherapy with anti-tumor immunity were published during this period. Around the mid-1970s was revived the notion that destruction of the tumor by radiation can stimulate anti-tumor immunity which will eradicate residual tumor cells [7, 8].

It was argued that this process could make the tumor its own cellular vaccine, and that it can be differentially modulated by different immune response-related treatments. This concept prompted a large volume of studies on the anti-tumor immune response after *in situ* destruction (ablation) of solid tumors in preclinical and clinical settings.

Tumor ablation is defined as the direct application of chemical, thermal, radiation, or electrical energy to a specific focal tumor in an attempt to achieve eradication or cytoreduction-inducing cellular necrosis. The major types are photon and particle radiation radiotherapy. To this effect, we developed a novel alpha radiation-based tumor treatment termed “Diffusing alpha emitters radiation therapy-DaRT,” which can cause fast tumor cell destruction within several days [9]. Thermal treatments such as radiofrequency (RFA), microwave, laser, ultrasound, and cryoablation. Electric-based treatments such as irreversible electroporation (IRE), tumor treating fields (TT Fields), or chemical and biological cytotoxic agents and photodynamic therapy. Apparently, any treatment modality, which destroys solid tumors *in situ*, can be considered as an ablative treatment [10, 11]. It is important to note that ablation of solid tumor foci can be performed instead or before surgery.

From 2000, it became increasingly apparent that in addition to radiotherapy, many standard cancer ablation methods may enhance the effectiveness of anti-tumor immune reactions, possibly due to increased inflammation, release of antigen and danger signals, immunogenic cell death pathways, and dampening the effects of regulatory cells. It was presented in a collection of papers [12].

## Ablation triggered anti-tumor immunity

A large variety of *in situ* tumor destruction techniques were reported to evoke specific anti-tumor immunity, which results in the elimination of residual malignant cells in primary tumors and distant metastases. The number of scientific reports per year on this topic increased 30-fold in the last 20 years. Anti-tumor immunity was reported to be triggered by treatment modalities such as radiation [13, 14], electric ablation [11], chemotherapy [15], thermal ablation, chemo- or radioembolization, irreversible electroporation, high-intensity focused ultrasound, and cryoablation [16].

What are the major immunological-related characteristics of tumor destruction *in situ* which result in triggering specific and innate anti-tumor immunity?

1. Tissue damage attracts inflammatory cells and causes the release of inflammatory cytokines
2. The extensive cell death releases large quantities of tumor-associated antigens in the context of danger-associated molecular patterns (DAMPs) to the immune system
3. Tumor antigens and DAMPs attract inflammatory and immune cells into the tumor vicinity and release the antigens to nearby lymph nodes
4. Elimination of the requirement to identify tumor-specific antigens for each patient
5. DNA damage will increase antigenicity and adjuvanticity. Radiation-inflicted DNA damage may produce immunogenic tumor-specific neoantigens and can generate aberrant nucleic acids to induce tumor immunogenicity [17, 18]

## Enforcement of anti-tumor immunity by immunomanipulation

Tumors evade or attenuate immune attack by a variety of complementary mechanisms of immunosuppression, loss of antigens, or loss of MHC molecules, which may operate in parallel. The presence of suppressive factors such as Treg cells or myeloid-derived suppressor cells (MDSC) in the tumor microenvironment and upregulation of surface ligands can mediate T-cell anergy (or exhaustion). Thus, the immune response triggered after ablation, which is mostly very weak, should be enforced by immunomodulating agents.

There are several types of measures which can be taken to promote anti-tumor immunity following *in situ* tumor ablation: (i) immune potentiation agents, (ii) agents to counteract suppressive mechanisms, and (iii) tumor vaccines and adoptive cell transfer.

i. Immunopotentiating agents such as adjuvants, dendritic cells, cytokines, and growth factors. [11, 14, 19, 20]
ii. Inhibitors of checkpoint molecules, regulatory T cells, and MDSC were also employed in combination with ablative procedures. Two recent reviews on the clinical outcomes of combination of radiotherapy and CPI claim that the results have been promising. The results, however, are quite limited, and refinement of the procedures is warranted [21]. In clinical trials with HCC patients, it was observed that the addition of RT helps to augment the effects of ICI [22]. Combination of thermal ablation may also be combined with CPI, and ongoing clinical trials were reported [23]
iii. Adoptive cellular therapy (ACT) is based on the patient’s immune cells, which are manipulated and then reinfused back into the patient. To date, there are several major modalities of ACT: tumor-infiltrating lymphocytes (TILs) which reside in the tumor and propagated by a cocktail of cytokines. Another type is genetically engineered T cell receptors (TCRs). TCRs are engineered T cells that recognize HLA-presented peptides derived from the proteins of all cellular compartments. A third type is chimeric antigen receptor (CAR) cells. CAR involves either T or NK lymphocytes engineered to carry a specific antigen-recognition receptor for cell surface antigens, independently of the major histocompatibility complex presentation. CARs recognize surface proteins typically through an antibody-derived scFv recognition domain
  Although ablation in combination with ACT is a promising approach, it did not yield yet sufficient promising results [20]. Recent studies have demonstrated that a combination of irreversible electroporation (IRE) and NK cell-based immunotherapy has the potential to improve patient survival in the advanced-stage liver and pancreatic cancers [24].

## Possible hazards of tumor ablation

No cancer treatment goes without a risk and tumor ablation may trigger tumor-promoting mechanisms. Radiotherapy, which is responsible for the abscopal effect, augments anti-tumor immunity, but may promote the number and function of immune-suppressive regulatory T cells [25]. This requires inhibition of Treg function in addition to immunotherapy with CPI.

It was also reported that incomplete RFA of a target tumor can sufficiently stimulate residual tumor cells to induce accelerated growth of distant tumors via the IL-6/c-Met/HGF pathway and VEGF production [26].

## Conclusions

There is a growing volume of data from both clinical and non-clinical studies that various modalities of *in situ* tumor destruction (ablation) can be a source of tumor antigens that otherwise would be difficult to expose to boost anti-tumor immune responses. The ensued immune reactivity can be further strengthened by both immune and non-immune modulators.

These findings prompt new ablation modalities which will increase the arsenal of the medical staff and promote cancer treatment.

What ablation method to use for optimal anti-tumor immunity stimulation?

Different ablative methods destroy tumor cells in different ways and affect differently the tumor microenvironment. This may impact the molecular entities presented to the immune response, the recruitment of immune cells, and the type of immune response. Thus, the resulting anti-tumor immunity may differ from one ablation modality to the other. The relative potency of different ablation treatments to reinforce anti-tumor immunity will be resolved with time with more clinical studies on this topic. Yet, the major factor in the decision which ablation method to use will be the eradication of the primary tumor. Nevertheless, immune stimulation by various ablation methods will be crucial for treatment of metastatic tumors and should be a factor in the decision of which one to use.

## Data Availability

All the information included in this article was published in scientific journals.
